# Melatonin Attenuates Ischemic-like Cell Injury by Promoting Autophagosome Maturation via the Sirt1/FoxO1/Rab7 Axis in Hippocampal HT22 Cells and in Organotypic Cultures

**DOI:** 10.3390/cells11223701

**Published:** 2022-11-21

**Authors:** Francesca Luchetti, Maria G. Nasoni, Sabrina Burattini, Atefeh Mohammadi, Marica Pagliarini, Barbara Canonico, Patrizia Ambrogini, Walter Balduini, Russel J. Reiter, Silvia Carloni

**Affiliations:** 1 Department of Biomolecular Sciences, University of Urbino Carlo Bo, 61029 Urbino, Italy; 2 Department of Cell Systems and Anatomy, Long School of Medicine, UT Health, San Antonio, TX 78229, USA

**Keywords:** autolysosome, autophagic flux, Forkhead box class O1, hippocampal slices, ischemia, Ras-related protein 7, silent information regulator 1

## Abstract

Dysfunctional autophagy is linked to neuronal damage in ischemia/reperfusion injury. The Ras-related protein 7 (Rab7), a member of the Rab family of small GTPases, appears crucial for the progression of the autophagic flux, and its activity is strictly interconnected with the histone deacetylase Silent information regulator 1 (Sirt1) and transcription factor Forkhead box class O1 (FoxO1). The present study assessed the neuroprotective role of melatonin in the modulation of the Sirt1/FoxO1/Rab7 axis in HT22 cells and organotypic hippocampal cultures exposed to oxygen-glucose deprivation followed by reoxygenation (OGD/R). The results showed that melatonin re-established physiological levels of autophagy and reduced propidium iodide-positive cells, speeding up autophagosome (AP) maturation and increasing lysosomal activity. Our study revealed that melatonin modulates autophagic pathways, increasing the expression of both Rab7 and FoxO1 and restoring the Sirt1 expression affected by OGD/R. In addition, the Sirt1 inhibitor EX-527 significantly reduced Rab7, Sirt1, and FoxO1 expression, as well as autolysosomes formation, and blocked the neuroprotective effect of melatonin. Overall, our findings provide, for the first time, new insights into the neuroprotective role of melatonin against ischemic injury through the activation of the Sirt1/FoxO1/Rab7 axis.

## 1. Introduction

Autophagy is a degradation and recycling process that is highly conserved in mammalian cells. Through the degradation of cytoplasmic organelles, proteins, and macromolecules, and the recycling of the breakdown products, autophagy plays an important role in cell homeostasis and supports their survival under stressful conditions. The autophagic process includes different stages that start with the formation of the autophagosome (AP), a double-membraned compartment that encloses the material to be recycled [1]. The AP then fuses its outer membrane with a lysosome to produce the autolysosome (AL). During fusion, the AP acidifies as it matures, obtaining the required hydrolytic enzymes for the subsequent autophagic degradation [2]. This process requires the movement of autophagosomes and late endosomes/lysosomes towards each other through the concerted actions of multiple regulators of membrane dynamics, which include the SNARE family proteins [3], the tethering proteins [4], and the Rab family’s small GTPases [5]. In particular, Rab small GTPases, which constitute the largest subfamily of the Ras superfamily, play critical roles in the various membrane trafficking steps, including vesicle formation, transport, docking, and fusion, and control of the progression of various membrane trafficking events, including endocytosis, exocytosis, and autophagy [6,7]. Among them, the Ras-related protein 7 (Rab7) appears crucial for the progression of the autophagic flux [5]. Rab 7 is predominantly detected in late endosomes [8], and its activity relies on its shuttling between the active (GTP-bound) and inactive state (GDP-bound) and vice versa [9]. The shuttling is regulated by three interrelated protein complexes, i.e., the guanine nucleotide exchange factors (GEFs), the GTPase-activating proteins, and the guanine nucleotide dissociation inhibitors (GDIs) [10]. To control the fusion events required for autophagosome maturation, Rab7 acts in concert with other players of the tethering and fusion machinery, such as specific members of the SNARE family [11,12] and the HOPS (homotypic fusion and protein sorting) complex [13], with the latter acting as a guanine nucleotide exchange factor for Rab7. Once activated, Rab7 mediates the attachment of the late-endosome/autophagosome to the dynein-dynactin complex through the recruitment of the Rab Interacting Lysosomal Protein (RILP), promoting the transport of AP towards the lysosomes for eventual fusion [14]. Changes in the expression and activity of Rab7 lead to an imbalance between autophagosome formation and degradation and are associated with the pathogenesis of various human diseases, including cancer, muscle diseases, and neurodegenerative disorders [15].

The effect of Rab7 on autophagosomal maturation and lysosomal fusion is strictly related to the activity of the class III histone deacetylase Sirt1 (silent information regulator 1) and its interaction with the Forkhead box class O (FoxO) family members FoxO1 and FoxO3 [16]. There is evidence that the activation of the Sirt1/FoxO1/Rab7 axis is required for preventing cell damage associated with dysfunctions of the autophagic processes [17]. For example, in breast cancer cells, loss of Sirt 1 causes Rab7-related lysosome activity dysfunction, resulting in the accumulation of larger multivesicular bodies [18], whereas, in cardiac myocytes, the synergic activity of Sirt1 and FoxO1-mediated starvation-induced autophagy through upregulation of Rab7, maintaining left ventricular function [17]. Thus, the role of the Sirt1/FoxO1/Rab7 axis in controlling autophagosomal dynamics appears crucial for improving cell survival and functionality.

Melatonin (N-acetyl-5-methoxytryptamine) possesses potent antioxidant, anti-inflammatory, and anti-apoptotic properties and has beneficial effects on a wide range of disorders [19], including brain ischemia [20,21,22]. Melatonin may regulate autophagy by directly modulating its activity and thus improving the proteolysis pathway [23,24] by indirectly reversing mitochondrial dysfunction caused by excessive oxidative stress or by reducing endoplasmic reticulum stress and hence the number of misfolded proteins [20,25,26]. Autophagy activation has a protective role during the neurodegenerative process linked to hypoxia-ischemia in neonatal rats and several pharmacological treatments, including melatonin [22,27], are effective in reducing brain injury in this acute neurodegenerative condition and also increasing autophagy [28,29].

In this study, we used oxygen-glucose deprivation followed by reoxygenation (OGD/R) of HT22 cells and organotypic hippocampal slice cultures as experimental models to assess the role of autophagy and the Sirt1/FoxO1/Rab7 axis as they relate to the neuroprotective effect of melatonin. We report here that the presence of melatonin during the reperfusion phase after OGD promotes early autophagosomal maturation in both HT22 cells and organotypic hippocampal slice cultures and that this effect is mediated by the Sirt1/FoxO1/Rab7 axis.

## 2. Materials and Methods

### 2.1. Cell Cultures

Hippocampal cells HT22 were cultured in DMEM-HAM’S F12, supplemented with 10% fetal calf serum, L-glutamine (100 mM), and 1% antibiotics (penicillin and streptomycin) (Sigma-Aldrich, St. Louis, MO, USA, P4333). The cells were incubated in a humidified 5% CO_2_ atmosphere at 37 °C. At 80% confluence, the cells were detached with trypsin-EDTA, washed, and sub-cultivated in new flasks for 1–2 days before the experiments.

### 2.2. Organotypic Hippocampal Slice Cultures

All animal procedures were performed in accordance with the Italian regulation for the care and use of laboratory animals (EU Directive 63/2010; D.L. 26/14). Organotypic hippocampal slice cultures were prepared as previously described in [30]. Briefly, 7-day-old Sprague-Dawley rat brains were removed and gently immersed in an ice-cold dissecting solution. Hippocampi were dissected on ice and cut into 400-μm thick transverse sections using a McIlwain Tissue Chopper. Slices with even margins and clear, uniform, well-defined pyramidal cell layers were selected. Slices were plated onto Millicell culture inserts (0.4 μm Millicell-CM, Sigma Aldrich, St. Louis, MO, USA, Z354996-50EA) pre-incubated with the culture medium (8.4 g/L MEM eagle medium, 20% horse serum heat inactivated, 30 mM HEPES, 1 mM CaCl_2_, 1 mM MgSO_4_, 1 mg/L insulin, 25% ascorbic acid solution, 13 mM D-glucose and 5.2 mM NaHCO_3_). After preparation, hippocampal cultures were maintained for 2 weeks in a 37 °C humidified incubator gasified with a 5% CO_2_-95% O_2_. The culture medium was changed three times per week.

### 2.3. Simulation of In Vitro Ischemia with Oxygen–Glucose Deprivation (OGD)

Hypoxia-ischemia was simulated by inducing transient oxygen-glucose deprivation followed by re-oxygenation (OGD/R) as previously described [31]. Briefly, HT22 cells were seeded at a density of 1 × 10^5^ cells/mL and incubated for 24 h to allow cells to adhere. Next, HT22 was maintained in the glucose-free culture medium and transferred into a temperature-controlled (37 °C) anaerobic chamber (Billups-Rothenberg Modular Incubator chamber, Billups-Rothenberg Inc., Del Mar, CA, USA; MIC-101) containing a gas mixture composed of 5% CO_2_-95% N_2_ for 8 h. Subsequently, the medium was replaced with normal DMEM containing glucose, and HT22 was returned to a normoxic condition for 2 or 18 h of reoxygenation in 5% CO_2_-95% O_2_ air. Controls were incubated with normal DMEM containing glucose in a humidified incubator with 5% CO_2_-95% O_2_ air at 37 °C for the same times as the OGD/R cultures.

In organotypic hippocampal slice cultures, hypoxia-ischemia was simulated by inducing transient OGD for 45 min followed by 2 h or 24 h of re-oxygenation as previously described [32]. Briefly, before anoxia, slice cultures were washed three times with a glucose-free slice culture medium and maintained in this medium up to the end of the OGD procedure. Slice cultures were then placed into a 2 L air-tight Billups-Rothenberg Modular Incubator chamber through which a 37 °C preheated 5% CO_2_-95% N_2_ gas was passed at 5–10 L/min. After 10 min gas flow, the chamber was sealed and placed in a 37 °C incubator for 35 min. Immediately after the OGD procedure, the culture tray was removed from the anoxic chamber, and the anoxic glucose-free slice culture medium was aspirated and replaced with a standard oxygenated culture medium. Slice cultures were maintained in this medium in a 37 °C incubator under 5% CO_2_-95% O_2_ re-oxygenation condition for 2 h or for 24 h for biochemical evaluations and cell damage assessment, respectively.

### 2.4. Drug Treatments

In HT22 cell experiments, melatonin (Sigma-Aldrich, St. Louis, MO, USA, M5250), dissolved in dimethyl sulfoxide (DMSO; Sigma-Aldrich, St. Louis, MO, USA, D5879) and diluted with a normal saline solution to a final concentration of 5% DMSO (vehicle) and a 50 μM dose were added to the medium immediately after the OGD procedure; thereafter, the cultures were maintained at 37 °C for 2 h or 18 h, as previously described [31]. In additional experiments, 1 μM EX-527 (Selisistat; Selleckchem.com, S1541) was added to OGD + Mel cells immediately after the OGD procedure and maintained at 37 °C for 2 h (OGD/R+Mel+EX−527) [33]. In the organotypic hippocampal slice culture experiments, melatonin (50 μM) was added to the culture medium immediately after the end of the OGD procedure and maintained in the medium for 2 or 24 h (OGD/R+Mel). The melatonin dose was chosen based on previous experiments that showed the protective effects of melatonin in organotypic hippocampal slice cultures [32]. An equivalent volume of the vehicle was added to the culture medium of both the Ctrl and OGD/R groups. In additional slice cultures, 100 μM EX-527 (Selisistat; Selleckchem.com, S1541) was added to Ctrl slices (Ctrl+EX−527) or 10 min before the OGD procedure in OGD+Mel condition (OGD/R+Mel+EX−527) [34].

### 2.5. Determination and Localization of Acid Organelles by LysoTracker Green-Uptake

LysoTracker Green (LTG; Molecular Probes, Eugene, OR, USA, L7526) is a fluorescent probe used for the determination and localization of acidic organelles in viable cells. It consists of a fluorophore linked to a weak base, partially protonated at a neutral pH, permeable to cell membranes, and typically concentrated in spherical organelles. LTG fluorescence measured by cytometry represents the overall mass of acidic organelles and reflects autophagic induction [35]. Cells were cultured at 37 °C and resuspended in a prewarmed (37 °C) medium containing 50 nM LysoTracker Green for 30 min. The green fluorescence was acquired by means of FACSCanto II flow cytometry (BD Biosciences, San Jose, CA, USA), collecting at least 10,000 events for each sample. Analyses were performed by using FACSDivaTM software. For confocal live imaging, cells were grown on MatTek glass bottom chambers (MatTek Corporation, Bratislava, Slovakia) at a density of 1 × 10^5^ cells/well. Following treatment, the cells were stained with LTG 50 nM for 30 min. The fluorescent images were captured by a confocal microscope (Leica TCS SP5 II Microsystem, Wetzlar, Germany). The images were further processed and analyzed in ImageJ 1.45 software (https://imagej.nih.gov/ij/, National Institutes of Health, Bethesda, MD, USA).

### 2.6. Immunofluorescence

Immunofluorescence experiments in HT22 cells were performed as previously described [31] and in organotypic hippocampal slice cultures, according to Gogolla et al. [36]. Briefly, organotypic hippocampal slice cultures, after treatment, were fixed for 5 min with 4% paraformaldehyde (pH 7.4) and then permeabilized for 24 h with 0.5% Triton X-100 in PBS at 4 °C. Next, the cultures were incubated for 24 h with blocking solution (20% BSA/PBS) and then incubated overnight at 4 °C with a polyclonal anti-Rab7 antibody (Cell Signaling Technology, EuroClone, Milan, Italy, #9367; 1:100) diluted in the blocking solution (5% BSA/PBS). In HT22 experiments, a monoclonal anti-LC3 antibody (Cell Signaling Technology, EuroClone, Milan, Italy, #2775; 1:200) diluted in a blocking solution (0.1% BSA/PBS) was also used. After being washed (3X) with PBS, the cultures were incubated for 3 h with FITC-conjugated anti-mouse or anti-rabbit secondary antibodies (Santa Cruz Biotechnology, Dallas, TX, USA, sc-516140, sc-2359; 1:100) or a PE-conjugated anti-rabbit secondary antibody (Novus Biologicals, Bio-Techne SRL, Milan, Italy, NB7581; 1:100). Subsequently, the samples were washed (3X) with PBS, and fluorescent images were captured by confocal microscope (Leica TCS SP5 II Microsystem, Wetzlar, Germany). Images were analyzed using NIH-Image J 1.45 software (https://imagej.nih.gov/ij/, National Institutes of Health, Bethesda, MD, USA).

### 2.7. Transmission Electron Microscopy (TEM)

HT22 cells were seeded in 75 cm^2^ flasks at a density of 2 × 10^6^ cells per well and allowed to adhere for 24 h. After treatment, the cells were washed, fixed, and embedded, as previously described [31]. The organotypic slices were immersed in 2.5% glutaraldehyde in 0.1 M phosphate buffer for 1 h. After two washes in 0.1 M of phosphate, the buffer cells were post-fixed for 1 h in 1% OsO_4_, dehydrated in a graded series of increasing concentrations of ethanol, and directly embedded on Millicell culture inserts (0.4 μm Millicell-CM, Sigma Aldrich, St. Louis, MO, USA, Z354996-50EA), in araldite at 60 °C. Subsequently, 1–2 µm thick sections were obtained from blocks using an LKB ultratome, stained with toluene blue for light microscopic examination. Additionally, 70 nm ultrathin sections were cut and put on 200 mesh nickel grids. Thin sections were counterstained with uranyless and lead citrate. Ultrastructural analysis was performed with a transmission electron microscope at 80 KV (Philips CM10) and imaged with an SIS MegaView II camera (Soft Imaging System GmbH, Münster, Germany) [37].

### 2.8. Western Blot Analysis

Protein extraction from HT22 cells was performed as previously described [31], and mitochondrial fractions were prepared according to Cantoni et al. [38]. Organotypic hippocampal slices were incubated on ice for 1 h with a lysis buffer (50 mM Tris, 5 mM ethylenediaminetetraacetic acid (EDTA), 150 mM NaCl, 0.5% Nonidet P-40, 1 mM phenylmethylsulfonyl fluoride, 1 mM sodium vanadate, and 1 mM sodium fluoride, pH 7.4) containing a protease inhibitor cocktail (Roche Italy, Monza, Italy 11836153001), lysed with a Sonicator Ultrasonic Liquid Processor XL (Heat System-Ultrasonics, Farmingdale, New York, NY, USA) and centrifuged at 21,500× *g* for 10 min at 4 °C to remove detergent-insoluble material. Supernatants were assayed for their protein concentration using the Bradford reagent (Sigma-Aldrich, St. Louis, MO, USA, B6916). Proteins from both experimental models were resolved (50 µg per lane) on 5–15% SDS-PAGE. After electrophoresis, proteins were electro-transferred to PVDF membranes using a semi-dry transfer apparatus. Membranes were incubated for 60 min at 4 °C in blocking solution (Tris-buffered saline containing 5% powdered milk and 0.1% Tween-20, pH 7.4) and probed overnight at 4 °C with the following primary antibodies: anti-LC3 (Cell Signaling Technology, EuroClone, Milan, Italy,#2775; 1:1000), anti-p62 (Sigma-Aldrich, St. Louis, MO, USA, P0067; 1 μg/mL), anti-Rab7 (Cell Signaling Technology, EuroClone, Milan, Italy,#9367; 1:1000), anti-Sirt1 (Cell Signaling Technology, EuroClone, Milan, Italy, #8469; 1:1000), anti-FoxO1 (MerkMillipore, Rome, Italy, 05-1075; 1:1000). The membranes were then incubated with horseradish peroxidase-conjugated anti-rabbit or anti-mouse antibodies for 1 h. The chemioluminescence (ECL, Amersham Pharmacia Biotech, Milan, Italy, GERPN2106) was detected using X-ray films that were scanned and analyzed using the NIH-Image J 1.45 software (https://imagej.nih.gov/ij/, National Institutes of Health, Bethesda, MD, USA). A primary mouse monoclonal antibody against β-actin (Santa Cruz Biotechnology, Dallas, TX, USA, sc-8432; 1:4000) was used as a loading control and for data normalization. Densitometric analyses were performed using the NIH-Image J 1.45 software (https://imagej.nih.gov/ij/, National Institutes of Health, Bethesda, MD, USA), and data were expressed as a percentage of control values.

### 2.9. Injury Assessment in HT22 Cells and Organotypic Hippocampal Slice Cultures

For organotypic hippocampal slice cultures, the cell damage was evaluated 24 h after the OGD procedure and melatonin treatment and assessed by image analysis of propidium iodide (PI, Sigma-Aldrich, St. Louis, MO, USA, P4170) uptake. PI is a polar compound that is not permeable to intact cell membranes; however, it penetrates damaged cells and binds to nuclear DNA to generate a bright red fluorescence. PI (5 µM) was added to the cultures two hours before the end of the incubation period with melatonin or the vehicle. PI incorporation into the slice cultures was assessed with an Olimpus BX-51 microscope (Olympus Italia S.r.l., Milan, Italy) using a standard rhodamine filter set (490/590 nm). Images were captured and analyzed using the NIH-Image J 1.45 software (https://imagej.nih.gov/ij/, National Institutes of Health, Bethesda, MD, USA). OGD-induced cell damage was calculated in each slice using the following expression:

Cell death (%) = Fd/Fo × 100(1)

where Fd is the fluorescence detected in the injured areas of the slice and Fo is the fluorescence detected in the whole slice. The PI fluorescence detected in hippocampal slice cultures treated with the glutamate receptor-agonist *N*-methyl-d-aspartic acid (NMDA, 10 µM, Sigma-Aldrich, St. Louis, MO, USA, M3262) for 4 h (calculated as described above) was considered 100% cell death. Results are reported as follows:

Cell death (%) = NMDA-induced cell death/OGD-induced cell death × 100(2)

For HT22 cell culture, supravital PI (Tali^®^ Viability Kit; Thermo Fisher Scientific, Milan, Italy) was assessed 2 h after the OGD procedure and melatonin treatment. Briefly, a 100 μL aliquot of cells (1 × 10^6^) from each sample was treated with 1 μg/mL PI for a few minutes and analyzed with Tali^®^ Image-Based Cytometer. The mean fluorescence was elaborated with the Floreada.io software and expressed as a fold of change compared to the control condition.

### 2.10. Statistical Analyses

Quantitative data are expressed as mean ± SD on the basis of at least three independent experiments. Differences between groups were analyzed using a one-way analysis of variance (one-way ANOVA), followed by a Newman-Keuls or Dunnett multiple comparison tests (Tukey post hoc test). A *p*-value ˂ 0.05 was considered significant. All statistical analyses were performed using GraphPad Prism 5.0 (GraphPad software, https://www.graphpad.com/).

## 3. Results

### 3.1. Melatonin Modulates Autophagy and Speeds up Autophagosomes Maturation during OGD/R in HT22 Cells

**Figure 1:**
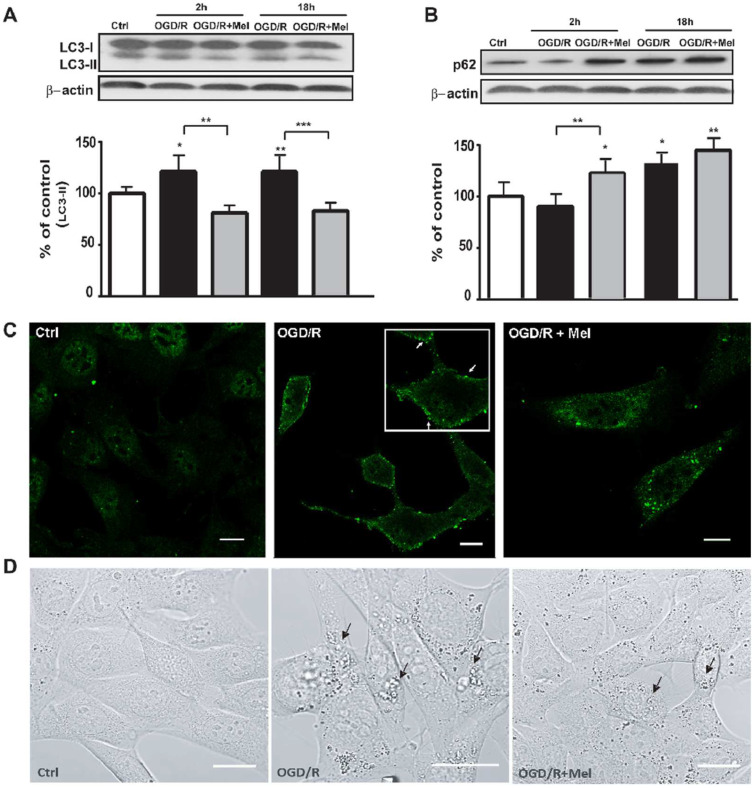
Melatonin modulates autophagic proteins during OGD/R in HT22 cells. (**A**) Representative Western blots and quantitative evaluations of LC3 II and p62 (**B**) expression in untreated HT22 cells (Ctrl), 8 h OGD-exposed cells followed by 2 or 18 h reoxygenation (OGD/R), and 8 h OGD-exposed cells followed by 2 h or 18 h 50 μmol/L melatonin reoxygenation (OGD/R + Mel). Data are normalized using the loading control β-actin and are expressed as percentages of control (mean ± SD (N = 3 independent experiments); * *p* < 0.05, ** *p* < 0.01 vs. Ctrl, one-way ANOVA followed by Dunnett multiple comparison test; ** *p* < 0.01, *** *p* < 0.01, Newman-Keuls multiple comparison test (lines). (**C**) Representative confocal images of LC3 immunostaining in Ctrl, OGD/R, and OGD/R + Mel cells after 18 h reoxygenation. The green puncta indicate LC3-positive structures (arrows). Scale bars: 10 μm. (**D**) Representative confocal microscope images of Ctrl, OGD/R, and OGD/R + Mel cells after 18 h reoxygenation. Arrows indicate vacuoles. Scale bars: 25μm.

The expression of LC3 was examined to assess the effect of OGD/R and melatonin on autophagy in HT22 cells; LC3 is an essential protein for the elongation and maturation of autophagosomes [39], and of the autophagic receptor p62/sequestosome-1 (SQSTM1), a protein that acts as a cargo receptor for the degradation of ubiquitinated proteins through autophagic or proteasomal pathways [40]. Immunoblot analyses showed that OGD/R significantly increased the expression of the lipidated form of LC3 (LC3 II). Melatonin reduced the increased LC3 II expression to control levels (Figure 1A) at both 2 and 18 h reoxygenation (OGD/R). The expression of p62 was slowly reduced compared with control levels 2 h after OGD/R (Figure 1B) but it increased after 18 h. The presence of melatonin in the reoxygenation phase increased the p62 expression over the control and the OGD/R levels at 2 h (Figure 1A,B), but its expression did not differ after 18 h (Figure 1B). To further support the activation of autophagy after 18 h reoxygenation we studied the cellular localization of LC3 with immunofluorescence experiments. The results showed that in the control condition LC3 was present in low amounts and located throughout the cytoplasm whereas in OGD/R cells we found many bright LC3-positive puncta around the membrane (Figure 1C). In the melatonin-treated cells, LC3-positive puncta were mainly located in the cytoplasm (Figure 1C) [1]. Brightfield microscope images revealed the presence of vacuoles in OGD/R cells (Figure 1D) that were in lower amounts after melatonin treatment (Figure 1D).

**Figure 2:**
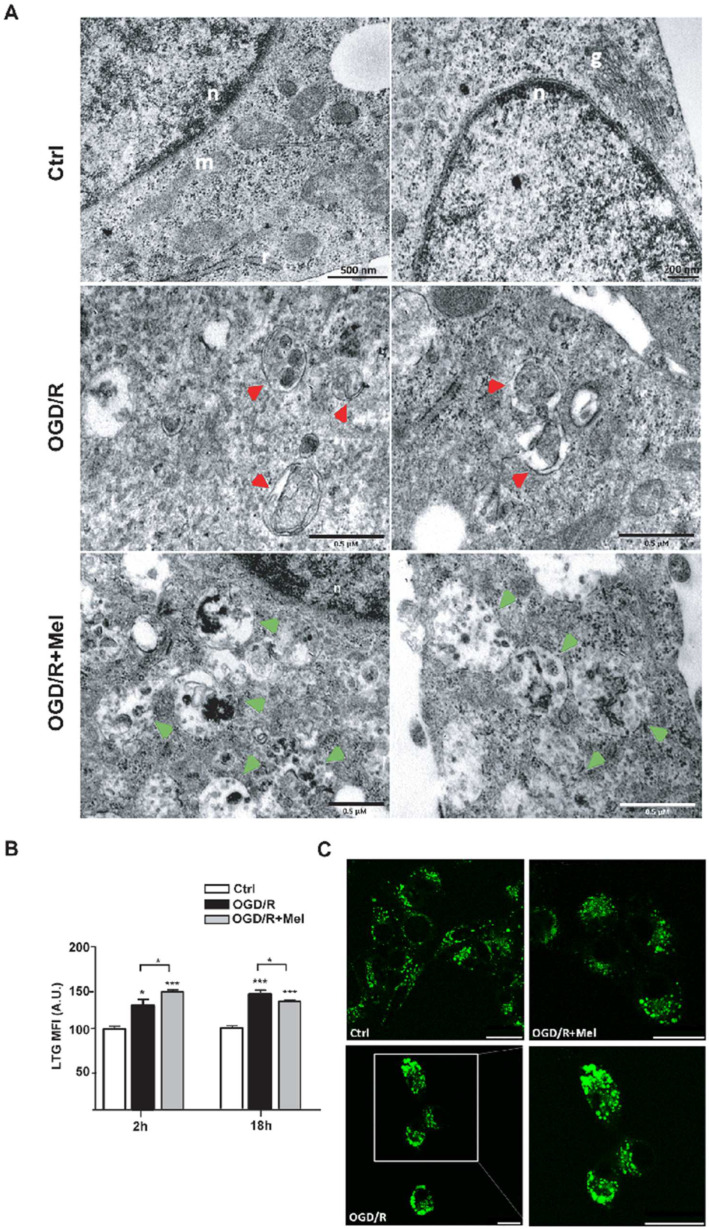
Melatonin accelerates autophagosomes maturation during OGD/R in HT22 cells. (**A**) Transmission electron microscopy (TEM) micrographs from untreated HT22 cells (Ctrl), 8 h OGD-exposed cells followed by 2 h reoxygenation (OGD/R), and 8 h OGD-exposed cells followed by 2 h 50 μmol/L melatonin reoxygenation (OGD/R + Mel). Images show autophagosomes (red arrowheads) and autolysosomes (green arrowheads) in OGD/R and OGD/R + Mel cells, respectively. n, nucleus; m, mitochondria; g, Golgi apparatus. (**B**) Quantitative evaluation and representative confocal microscopy images (**C**) of Lysotracker green (LTG) labeling in Ctrl, OGD/R, and OGD/R + Mel cells after 2 or 18 h reoxygenation. Results are expressed as mean fluorescence intensity (MFI) (mean ± SD, N = 3 independent experiments performed in triplicate); * *p* < 0.05, *** *p* < 0.001, vs. Ctrl, one-way ANOVA followed by Tukey’s multiple comparison test. Scale bars: 25 μm.

To get more insight on the nature of these vacuoles, we used transmission electron microscopy (TEM), which represents the gold standard for studying autophagy. TEM/ultrastructural analysis was performed after 2 h in the OGD/R and OGD/R + Mel conditions. This time point was chosen because we were interested in examining the early stages of autophagy activation. Results showed the presence of autophagic vacuoles (AVs) in both OGD/R and OGD/R + Mel cells (Figure 2A). In the OGD/R condition, autophagosomes (APs) appeared in an early phase autophagy process with a clear double-limiting membrane separated by a small space, and full-cargo vacuoles with enclosed cytoplasmic material not yet degraded, including electron-dense ribosomes (Figure 2A, red arrowheads). In contrast, in OGD/R + Mel cells, autophagic vacuoles appeared partially degraded together with the material inside. In addition, the presence of electron-dense lysosomal aggregates highlighted the presence of autolysosomes (ALs; Figure 2A, green arrowheads), which were not evident in the OGD/R condition (Figure 2A).

Using flow cytometry analysis and fluorescence confocal microscopy analysis of the acidotropic dye Lysotracker Green (LTG), we evaluated the lysosome content. Flow cytometry analysis showed increased LTG fluorescence intensity in OGD/R cells compared to control cells after 2 h or 18 h reoxygenation (Figure 2B). LTG labeling was significantly increased in OGD/R + Mel cells compared with both control and OGD/R conditions 2 h after the injury (Figure 2B). However, after 18 h reoxygenation, LTG labeling in OGD/R + Mel cells was slightly but significantly reduced compared with OGD/R cells (Figure 2B). Confocal image analysis performed after 18 h reoxygenation confirmed the larger number of LTG-positive puncta in OGD/R cells that was reduced after melatonin treatment (Figure 2C).

### 3.2. Melatonin Modulates the Sirt1/FoxO1/Rab7 Axis Involved in Autophagosomes Maturation during OGD/R in HT22 Cells

**Figure 3:**
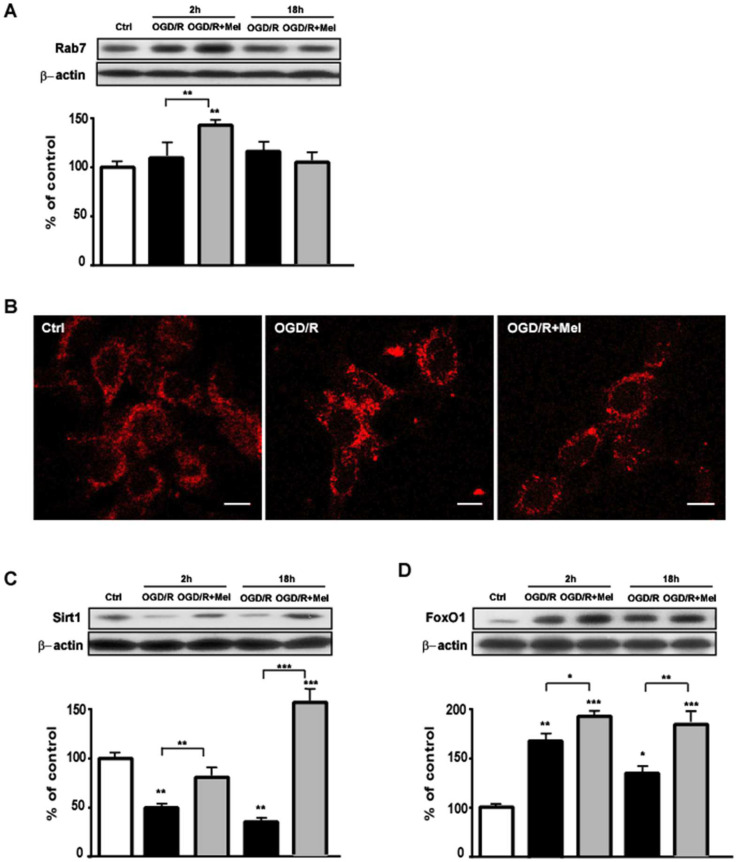
Melatonin modulation of the Sirt1/FoxO1/Rab7 axis during OGD/R in HT22 cells. (**A**) Representative Western blot and quantitative evaluation of Rab7 expression in untreated HT22 cells (Ctrl), 8 h OGD-exposed cells followed by 2 or 18 h reoxygenation (OGD/R), and 8 h OGD-exposed cells followed by 2 or 18 h 50 μmol/L melatonin reoxygenation (OGD/R + Mel). Data are normalized to the loading control β-actin and are expressed as percentages of control (mean ± SD; N = 3 independent experiments); ** *p* < 0.01 vs. Ctrl, one-way ANOVA followed by Dunnett multiple comparison test; ** *p* < 0.01, Newman-Keuls multiple comparison test (lines). (**B**) Representative confocal images of Rab7 immunostaining in Ctrl, OGD/R, and OGD/R + Mel cells after 18 h reoxygenation. The red dots indicate Rab7 on late endosomes. Scale bars: 10 μm. (**C**) Representative Western blots and quantitative evaluation of Sirt1 and FoxO1 (**D**) expression in Ctrl, OGD/R, and OGD/R + Mel cells after 2 h or 18 h reoxygenation. Data are normalized to the loading control β-actin and are expressed as % of control (mean ± SD, N = 3 independent experiments); * *p* < 0.05, ** *p* < 0.01, *** *p* < 0.001 vs. Ctrl, one-way ANOVA followed by Dunnett multiple comparison test; * *p* < 0.05, ** *p* < 0.01, *** *p* < 0.001, Newman-Keuls multiple comparison test (lines).

The data reported above suggest that melatonin promotes autophagosome maturation. As the small GTPase protein Rab7 is crucial for the progression of the autophagic flux [5,41,42], we tested whether the prompt autophagosome maturation and lysosome fusion observed after melatonin involved the Rab7 protein. Figure 3A shows that Rab7 expression was slightly but not significantly increased at both 2 h and 18 h after OGD/R. Rather, Rab7 was significantly increased after 2 h reoxygenation in the OGD/R + Mel condition (Figure 3A) and returned to control levels at 18 h (Figure 3A). Confocal immunofluorescence analysis performed after 18 h reoxygenation showed punctate labeling of the cytoplasmic Rab7 protein in all experimental conditions. However, in OGD/R conditions, cells exhibited Rab7 accumulation in the perinuclear region, whereas, in OGD/R + Mel cells, the protein had a more general cytoplasmic distribution (Figure 3B).

The deacetylase Sirt1 and the transcription factor FoxO1 are upstream of Rab7 in autophagy activation [16]. Since Sirt1 is rapidly induced by melatonin after hypoxia-ischemia-induced brain injury [22], we assessed the possible involvement of these proteins under the current experimental conditions. Figure 3C shows that the expression of Sirt1 was significantly reduced in OGD/R cells at both 2 and 18 h after the insult (*p* ≤ 0.01 vs. Ctrl; Figure 3C). In the presence of melatonin, Sirt1 was almost completely restored to control values after 2 h reoxygenation and was overexpressed after 18 h reoxygenation (*p* ≤ 0.001 vs. Ctrl; Figure 3C). FoxO1 was significantly increased after OGD/R compared with the Ctrl condition (Figure 3D) and further enhanced in the presence of melatonin at both 2 h and 18 h (Figure 3D).

**Figure 4:**
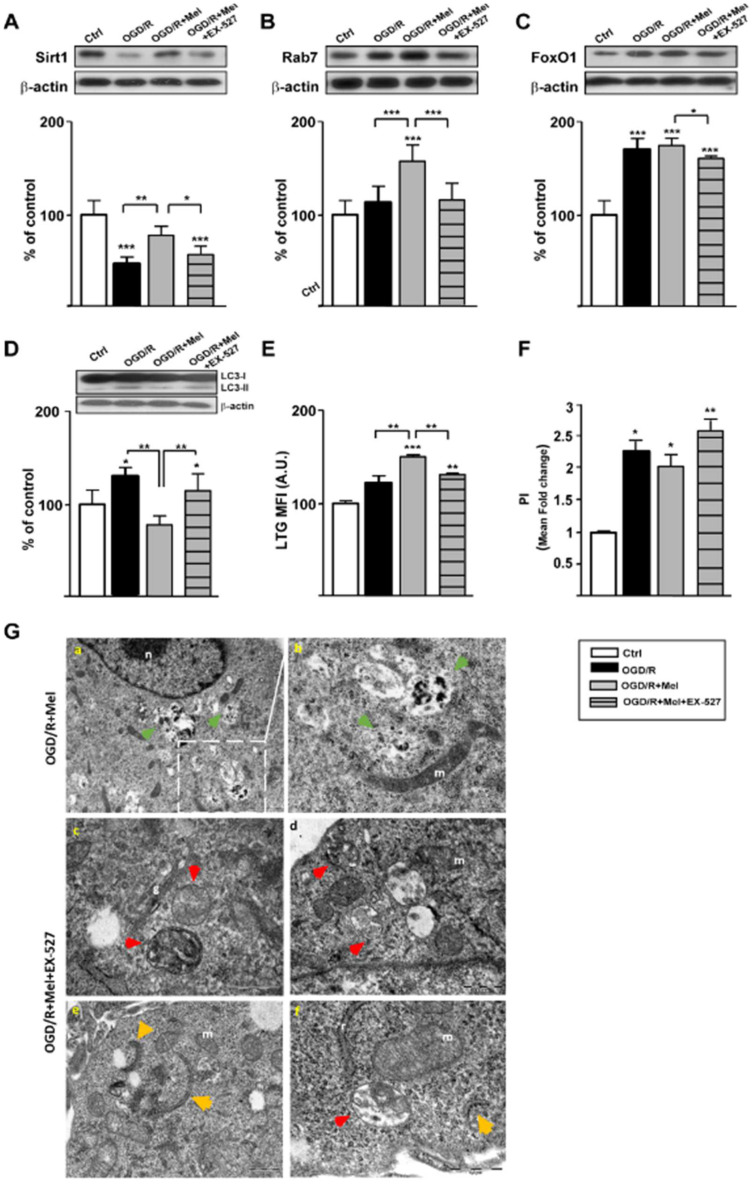
Effect of the Sirt1 inhibitor EX-527 on Sirt1/FoxO1/Rab7 axis and lysosome formation during OGD/R in HT22 cells. (**A**) Representative Western blots and quantitative evaluation of Sirt1, Rab7 (**B**), FoxO1 (**C**), and LC3 II (**D**) expression in untreated HT22 cells (Ctrl), 8 h OGD-exposed cells followed by 2 h reoxygenation (OGD/R), 8 h OGD-exposed cells followed by 2 h 50 μmol/L melatonin reoxygenation (OGD/R + Mel) and EX-527 10μmol/L-treated cells exposed to 8 h OGD followed by 2 h 50 μmol/L melatonin and reoxygenation (OGD/R + Mel + EX-527). Data are normalized to the loading control β-actin and are expressed as % of control (mean ± SD, N = 3 independent experiments); * *p* < 0.05, *** *p* < 0.001 vs. Ctrl, one-way ANOVA followed by Dunnett multiple comparison test; * *p* < 0.05, ** *p* < 0.01, *** *p* < 0.001, Newman-Keuls multiple comparison test (lines). (**E**) Quantitative evaluation of Lysotracker green (LTG) labeling in Ctrl, OGD/R, OGD/R + Mel and OGD/R + Mel + EX-527 cells after 2 h reoxygenation. Results are expressed as mean fluorescence intensity (MFI) (mean ± SD, N = 3 independent experiments performed in triplicate); * *p* < 0.05, ** *p* < 0.01, *** *p* < 0.001 vs. Ctrl, one-way ANOVA followed by Tukey’s multiple comparison test. (**F**) Quantitative evaluation of cell death by Propidium Iodide (PI) fluorescence analysis in Ctrl, OGD/R, OGD/R + Mel and OGD/R + Mel + EX-527 cells after 2 h reoxygenation. * *p* < 0.05, ** *p* < 0.01 vs. Ctrl, one-way ANOVA followed by Tukey’s multiple comparison test. (**G**) Transmission Electron Microscopy (TEM) micrographs from OGD/R + Mel and OGD/R + Mel + EX-527 cells after 2 h reoxygenation. Images show autolysosomes (green arrowheads) in OGD/R + Mel cells (panels **a**,**b**) and phagophores (orange arrowheads) and autophagosomes (red arrowheads) in OGD/R + Mel + EX-527 cells (panels **c**–**f**). n, nucleus; m, mitochondria; r, endoplasmic reticulum; g, Golgi apparatus.

To assess the relevance of the Sirt1/FoxO1/Rab7 axis in the protective effect of melatonin, we used the selective Sirt1 inhibitor EX-527 [43]. In agreement with the information reported above, in this set of experiments, we found the same trend of expression for Sirt1, Rab7, and FoxO1 after OGD/R and melatonin treatment. The addition of EX-527 to the OGD/R + Mel condition significantly reduced the increased Sirt1 expression (Figure 4A) as well as the increased expression of Rab7 and FoxO1 (Figure 4B,C). EX-527 also reduced both the increased expression of LC3 II (Figure 4D) and the increased LTG labeling induced by melatonin in the early phase of autophagy activation (Figure 4E) and enhanced the number of PI-positive cells (Figure 4F). Furthermore, TEM revealed that EX-527 also affected melatonin-induced ALs formation, as indicated by the presence of lysosome and ALs in OGD/R + Mel that was lacking in OGD/R + Mel + EX-527 condition (Figure 4G, panels a and b; green arrowheads). Additionally, in OGD/R + Mel + EX-527 cells, several phagofores and APs structures were apparent (Figure 4G, panels c–f; orange and red arrowheads, respectively).

### 3.3. Melatonin Promotes Autophagosomal Maturation through the Activation of Sirt1/FoxO1/Rab7 Axis during OGD/R in Organotypic Hippocampal Slice Cultures of Neonatal Rats

**Figure 5:**
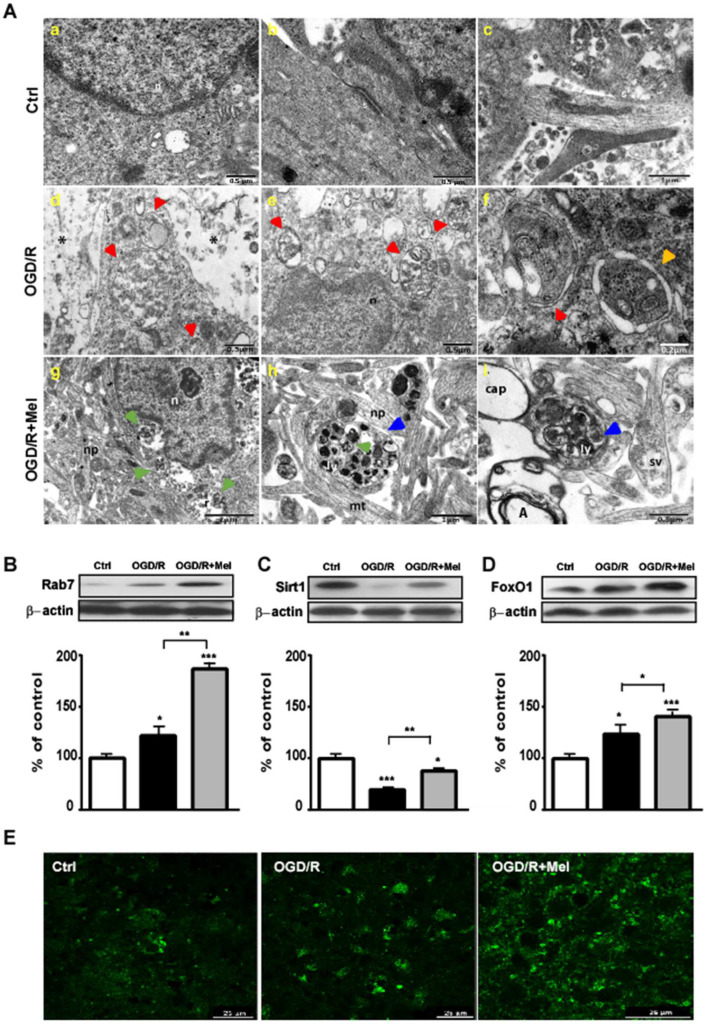
Melatonin modulates the Sirt1/FoxO1/Rab7 axis during OGD/R in organotypic hippocampal slice cultures. (**A**) Transmission electron microscopy (TEM) micrographs from untreated hippocampal slice cultures (Ctrl, panels **a**–**c**), 45 min OGD-exposed hippocampal slice cultures followed by 2 h reoxygenation (OGD/R, panels **d**–**f**), 45 min OGD-exposed hippocampal slice cultures followed by 2 h 50 μmol/L melatonin reoxygenation (OGD/R + Mel, panels **g**–**i**). Images show autophagosomes (red arrowheads) and autolysosomes (green arrowheads) in OGD/R and OGD/R + Mel cells, respectively. n, nucleus; s, synapse; np, neuropil, mt, microtubule; ly, lysosome; sv, synaptic vesicles; cap, capillary; A, Axon; *, necrotic area; orange arrowhead, phagophore; blue arrowhead, dystrophic neurite. (**B**) Representative Western blot and quantitative evaluation of Rab7, Sirt1 (**C**) and FoxO1 (**D**) in Ctrl, OGD/R, and OGD/R + Mel hippocampal slice cultures after 2 h reoxygenation. Data are normalized to the loading control β-actin and are expressed as a percentage of control (mean ± SD (N = 3 independent experiments); * *p* < 0.05, *** *p* < 0.001 vs. Ctrl, one-way ANOVA followed by Dunnett multiple comparison test; * *p* < 0.05, ** *p* < 0.01, Newman-Keuls multiple comparison test (lines). (**E**) Representative confocal images of Rab7 immunostaining in Ctrl, OGD/R, and OGD/R + Mel hippocampal slice cultures after 2 h reoxygenation. The green dots indicate Rab7 on late endosomes. Scale bars: 25, 50 μm.

To strengthen the experimental evidence reported above, we analyzed autophagy activation and the Sirt1/FoxO1/Rab7 axis after OGD/R in organotypic hippocampal slice cultures; these represent an ex vivo experimental model with tissue organization comparable to the brain tissue [44]. In control slices, TEM analysis revealed the intact envelope of the nucleus and preservation of microtubules and pre- and post-synaptic membranes (Figure 5A, panels a–c). After OGD/R, we observed lower tissue integrity (Figure 5A, panel d, asterisks) with autophagosomes showing double membranes containing partially degraded cytoplasmic material (Figure 5A, panels d and f; red arrowhead). Higher magnification images showed phagophores with enlarged double membranes containing fragments of cytoplasmic organelles (Figure 5A, panel f, orange arrowhead). In the presence of melatonin during the reoxygenation phase, we found an improved ultrastructural preservation of axonal and dendritic processes in neuropils and microtubules (Figure 5A, panel g and h), as well as the preservation of synaptic connections with their associated vesicles (Figure 5A, panel i). In contrast to the OGD/R condition, in OGD/R + Mel hippocampal slices, we observed the presence of autolysosomes containing partially degraded material (Figure 5A, panels g–i, green arrowhead). A higher magnification analysis revealed the presence of dystrophic neurites with a condensed axoplasmic matrix and extensive accumulation of autophagic vacuoles with amorphous electron dense material representing autolysosomes and lysosomes (Figure 5A, panels h and i; blue arrowheads).

We also studied the Sirt1/FoxO1/Rab7 axis in hippocampal slice cultures. In the control slices, Rab7 was expressed in low amounts. In the OGD condition, followed by 2 h reoxygenation, Rab7 expression was significantly increased (Figure 5B) and was further remarkably elevated over the OGD/R condition when melatonin was present during the reoxygenation phase (Figure 5B). Rab7 modulation was confirmed by immunohistochemical analysis. Low Rab7 expression was detected in pyramidal-like neuronal cells of control hippocampal slices (Figure 5E). The number of Rab7-positive cells, however, were appreciably greater in hippocampal slices exposed to OGD/R (Figure 5E) and markedly increased after melatonin treatment (Figure 5E). Figure 5 also shows that Sirt1 expression was significantly reduced after OGD/R and its expression was almost completely restored in the presence of melatonin (Figure 5C). In addition, melatonin enhanced FoxO1 expression over the control and OGD/R conditions (Figure 5D).

**Figure 6:**
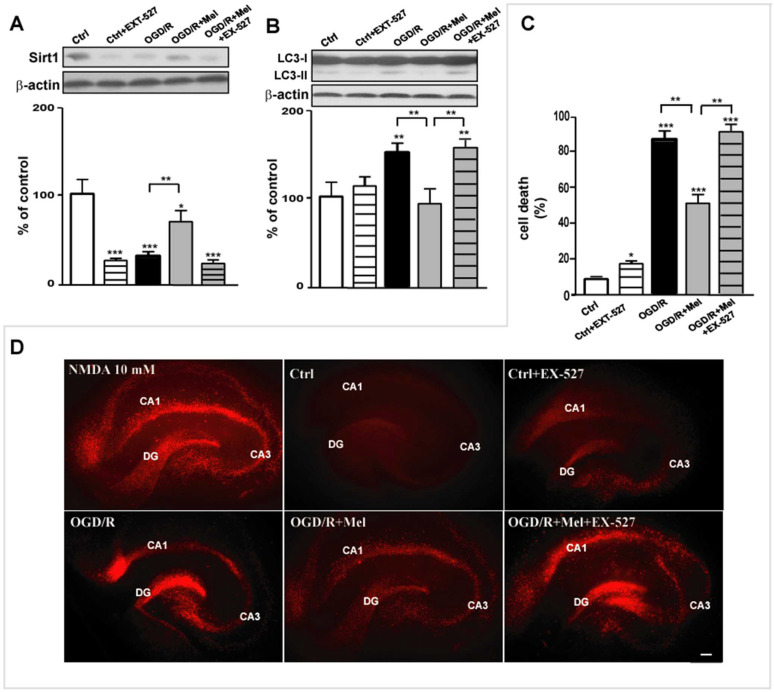
Sirt1 inhibitor EX-527 blocks the melatonin effects on OGD/R-induced cell death in organotypic hippocampal slice cultures. (**A**) Representative Western blot and quantitative evaluation of Sirt1 and LC3 II (**B**) expression in vehicle-treated (Ctrl), EX-527-treated (Ctrl + EX-527) hippocampal slice cultures, 45 min OGD-exposed hippocampal slice cultures followed by 2 h reoxygenation (OGD/R), 45 min OGD-exposed hippocampal slice cultures followed by 2 h 50 μmol/L melatonin reoxygenation (OGD/R + Mel), and EX-527-treated and 45 min OGD-exposed hippocampal slice cultures followed by 2 h 50 μmol/L melatonin reoxygenation (OGD/R + Mel + EX-527). Data are normalized to the loading control β-actin and are expressed as % of control (mean ± SD, N = 3 independent experiments); * *p* < 0.05, ** *p* < 0.01, *** *p* < 0.001 vs. Ctrl, One-way ANOVA followed by Dunnett multiple comparison test; ** *p* < 0.01, Newman-Keuls multiple comparison test (lines). (**C**) Quantitative evaluation of cell death and representative images (**D**) of Propidium Iodide (PI) fluorescence from NMDA-treated (NMDA 10 μM), Ctrl, Ctrl + EX-527, OGD/R, OGD/R + Mel and OGD/R + Mel + EX-527 hippocampal slice cultures after 24 h reoxygenation. * *p* < 0.05, *** *p* < 0.001 vs. Ctrl, one-way ANOVA followed by Dunnett multiple comparison test; ** *p* < 0.01, Newman-Keuls multiple comparison test (lines), *n* = 15/group. CA1, DG (dentate gyrus), and CA3 represent the 3 main areas of the hippocampus damaged by NMDA or OGD/R and subjected to quantitative analysis. Images were acquired as described in Materials and Methods. Scale bar, 50 μm.

In line with the experiments performed in HT22 cells, in hippocampal slices the Sirt1 inhibitor EX-527 completely blocked the effects of melatonin on Sirt1 expression (Figure 6A) as well as on LC3 II expression that returned to OGD/R levels (Figure 6B). To study the consequences of this inhibition, we evaluated EX-527 on hippocampal injury. As shown in Figure 6, PI labeling in control hippocampal slices was low after 24 h reoxygenation (Figure 6C,D). OGD/R caused a marked increase of PI labeling, particularly in the CA1-CA3 and DG areas of the hippocampus, indicating extensive cell death in these areas (Figure 6C,D). PI labeling was significantly reduced when melatonin was present during the reperfusion phase (Figure 6C,D). EX-527 increased PI labeling in control conditions (Figure 6C,D) and completely negated the protective effect of melatonin, causing an increase in the percentage of cell death from 47.4% ± 4.5% to 84.3% ± 2.6% (Figure 6B).

## 4. Discussion

Autophagy is a tightly orchestrated cellular process aimed at maintaining cellular homeostasis and function in both physiological and stressful conditions [45]. The role of autophagy in cerebral ischemia/reperfusion injury is still widely debated and controversial [46], although, undoubtedly, autophagy activation plays an important role in the pathologic process [47], and in the neuroprotective effect of preconditioning and pharmacological treatments [28,48]. Herein, we assessed the role of autophagy in the neuroprotective effect of melatonin using two models of in vitro ischemia simulated by OGD and reoxygenation. We show that in both models melatonin re-established physiological levels of autophagy affected by OGD and promoted autophagosome maturation via the Sirt1/Foxo1/Rab7 pathway. Blocking APs maturation also prevented the protective effect of melatonin.

To assess autophagy activation, we assessed the expression of LC3 II, LC3 localization, and the expression of p62. We found different patterns of LC3 II and p62 expression 2 h after OGD/R, showing autophagy activation. Eighteen hours after OGD/R, however, despite a clear increase in LC3 II, p62 was not decreased; nevertheless, immunohistochemical experiments showed LC3 puncta around the cell membrane indicating that autophagy is still activated [39]. Our findings are in line with previous results by Jain et al. in a model of starvation-induced autophagy. These authors reported that p62 was reduced by autophagic degradation early during starvation but that they were restored to basal levels upon prolonged starvation. The different effect on p62 has been explained by the fact that the protein works as a selective autophagy receptor in the early phase of injury and as a member of the protein battery induced by Nrf2 in response to oxidative stress under conditions of prolonged cellular stress [49]. Similar findings were also reported by Duran et al., who showed that autophagy-induced starvation was associated with p62 transcriptional synthesis [50]. Altogether, these findings show that the expression of p62 does not always inversely correlate with autophagy activation.

Autophagy is a highly dynamic and actively modulated multi-step process where each step exerts different functions in a variety of cellular contexts, making autophagy multifunctional [51]. To perform an accurate interpretation of autophagy, multiple methods allowing for the estimation of the overall autophagic flux are required as opposed to merely measuring the steady-state levels of autophagy proteins [39]. In particular, the presence of single or double membranes, electron dense content, or degraded organelles can discriminate early phagophores and autophagosomes from mature autophagolysosomes. We therefore supported the quantitative analysis of the autophagic markers LC3 II and p62 after OGD/R and melatonin with TEM. TEM is recognized as the only tool that can be used to reliably identify the morphology of autophagic structures at a nm range resolution, as they exist in the normal cellular environment and location among all other cellular components; only this procedure allows for their precise identification [39]. In the OGD/R condition for HT22 cells, TEM analysis revealed the presence of APs during the early phase of the ischemic damage, i.e., after 2 h reoxygenation. Conversely, at the same time point in the OGD/R + Mel condition we found numerous ALs that were not apparent when melatonin was absence. In keeping with these observations, in melatonin-treated cells we also observed higher lysosomal content and reduced expression of the lipidated LC3. The latter is usually associated with newly forming autophagosomal membranes, where it remains anchored until autophagosomes fuse with lysosomes [52].

To assess the relevance of the in vivo findings we used organotypic hippocampal slice cultures, which represent an ex vivo experimental model that has a tissue organization comparable to the intact hippocampus [44]. This model preserves the interaction between neurons and glial cells, necessary for supporting the energetic status of neurons under ischemic conditions [53]. In keeping with the results obtained in HT22 cells, we found that melatonin preserved the hippocampal tissue integrity affected by OGD/R and induced ALs formation; this finding provides further evidence that melatonin-induced neuroprotection involves the promotion of Aps maturation. It is well documented that dysfunctional AP maturation is associated with various human diseases, including neurodegenerative disorders, cancer, and muscle diseases [54]. Accumulation of APs within cells is associated with the degeneration of ischemic neurons [55]; thus, fostering AP maturation may represent a potential target for therapeutic interventions [56,57]. In addition, it has reported that hypoxic preconditioning provides neuroprotection against transient global cerebral ischemia in adult rats by enhancing AP maturation [58]. Antioxidants reduce dopamine toxicity for hippocampal neurons and restore AP formation in these cells [59].

AP maturation and the subsequent fusion with endosomes/lysosomes requires the coordinated actions of several multiple regulators of membrane dynamics, such as SNAREs, tethering proteins, and Rab GTPases [3,4,5]. Here we found that OGD/R increased the expression of the GTPase Rab7, a crucial factor for the progression of the autophagic flux [5,41,42]; melatonin further increased Rab7 expression in the early phase of ischemic damage (2 h after OGD/R) in both HT22 cells and organotypic hippocampal cultures. Rab 7 increase is concomitant with a higher lysosomal content, early formation of autophagolysosomes, and reduction of the lipidated LC3 expression. Rab 7 expression returned to control values at 18 h, suggesting that the protein is mainly involved in the early phase of autophagy activation. All these effects are associated with a reduced number of PI-positive cells evaluated 18 h after OGD/R. In mammalian cells, Rab 7 is involved in the regulation of AP formation, and it is essential for the AP–AL fusion and lysosomal genesis [41,60,61]. Its inactivation or deficiency causes the disruption of lysosome biogenesis, inhibition of autophagic vacuoles fusion, and APs accumulation [62,63]. Mutations in the Rab7 gene or dysfunction of the Rab7 protein and Rab7-interacting effectors may contribute to diseases, including neurological diseases [64,65] and cancer [66]. Rab7 knockout mice showed an explicit late buildup of neuronal endosomes/autophagosomes, which leads to neurodegeneration [67]. In addition, the suppression of the transcription of Rab7 gene, induced by the hypoxic-ischemic insult, caused impaired autophagosome clearance in murine cardiomyocytes and consequent cell death [68]. Rab7 involved in AP maturation contributes to neuroprotection after hypoxic preconditioning in global cerebral ischemia [58] and subarachnoid hemorrhage injury [69] in adult rats. In line with this evidence, we suggest that Rab7 mediated early AP maturation contributes to melatonin neuroprotective effect in OGD/R condition.

To the best of our knowledge, this is the first evidence showing that melatonin promotes AP maturation through Rab7 overexpression in ischemic hippocampal cells. Interestingly, the effects of melatonin are strictly related to the rescue of Sirt1 and concurrent overexpression of FoxO1. The Sirt1 inhibitor EX-527 blocked FoxO1 and Rab7 overexpression as well as the AL formation induced by melatonin, indicating that activation of Sirt1 is upstream to the AP maturation and appears essential for neuroprotection. Consistent with our finding, it has been previously reported that Sirt1 is required for autophagy activation [68]. Indeed, Sirt1 directly affects autophagy by deacetylating the products of autophagy-related proteins ATG5, ATG7, and ATG8 [70]. In addition, Sirt1 is also directly involved in AP maturation, through its interaction and consequent deacetylation of cortactin, which is required for autophagosome-lysosomes fusion [71,72]. Sirt1 is also involved in autophagy control through the deacetylation of FoxO transcription factors [73], which have multifaceted roles in autophagy regulation and dysregulation, including the control of the AP maturation and their fusion with lysosomes [74]. Here, we found that melatonin restored OGD/R-induced Sirt1 depletion, which was accompanied by FoxO1 overexpression. The mechanisms that underlie the overexpression of FoxO1 by melatonin requires further investigation as does the balance between acetylation and deacetylation of FoxO1 by Sirt1, especially considering the new evidence of a crucial role of FoxO-autophagy axis in cell death and survival [74].

Although in this study we used both in vitro and ex vivo models, it should be considered that the in vivo situation is more complex, as, in the hippocampus, there are different cell types that cannot be represented in our models, such as the vascular cells with their interactions with the blood flux and the immune cells.

In summary, our findings document that melatonin attenuated ischemic-like cell injury by promoting AP maturation via the Sirt1/FoxO1/Rab7 axis. We suggest that the rapid activation of Sirt1 induced by melatonin, as observed here and in our previous work [22], supports cell survival during ischemia, and also hastens the formation of autolysosomes, and improves protein recycling. Our present data further support the idea that autophagy is a component of pro-survival signaling that promotes the recovery of injured cells [29,75].

## Data Availability

Not applicable.

## Abbreviations

AL	autolysosome
AP	autophagosome
FoxO1	Forkhead box class O1
Mel	melatonin
OGD/R	oxygen-glucose deprivation/reoxygenation
Rab 7	Ras-related protein 7
Sirt1:	Silent information regulator 1
